# Thrombin generation, viscoelastic coagulation profiles, and disease severity in critically Ill COVID-19 patients: a prospective observational study

**DOI:** 10.1186/s12871-026-04166-3

**Published:** 2026-08-13

**Authors:** Florian Espeter, Lena Garczarek, David Künne, Henning Kuhlmann, Stefan Heitmeier, Thomas Schmoch, Thorsten Brenner, Karsten Schmidt

**Affiliations:** 1 Department of Anesthesiology and Intensive Care Medicine, University Hospital Essen, University Duisburg-Essen, Essen, 45147 Germany; 2 Bayer AG, Research and Development Pharmaceuticals, Wuppertal, 42117 Germany; 3 Departement of Anesthesiology and Intensive Care Medicine, Hôpitaux Robert Schuman- Hôpital Kirchberg, 9, Rue Edward Steichen, Luxembourg City, 2540 Luxembourg

**Keywords:** COVID-19, Thrombin generation, Rotational thromboelastometry, Coagulation, COVID-19-associated coagulopathy, Disease severity, Extracorporeal membrane oxygenation

## Abstract

**Background:**

COVID-19-associated coagulopathy is characterized by complex alterations in haemostasis. While thrombin generation assays (TGA) provide a global assessment of coagulation potential, data on thrombin generation in critically ill COVID-19 patients, particularly those requiring extracorporeal membrane oxygenation (ECMO), remain limited.

**Objective:**

To describe thrombin generation, viscoelastic coagulation profiles, and contact pathway-related markers in critically ill COVID-19 patients and to explore their association with disease severity during the first week of intensive care treatment.

**Methods:**

In this prospective single-center observational study, 48 critically ill patients with COVID-19-associated acute respiratory distress syndrome were included. TGA parameters, rotational thromboelastometry (ROTEM), and contact pathway-related markers were assessed on days 1, 3, and 7 after ICU admission. Associations with disease severity were explored using the Sequential Organ Failure Assessment (SOFA) score. Analyses were exploratory and primarily descriptive.

**Results:**

Endogenous thrombin potential (ETP) was inversely correlated with SOFA score on day 1 (*r* = − 0.43), day 3 (*r* = − 0.49), and day 7 (*r* = − 0.31). Peak thrombin showed similar inverse correlations on days 1 and 3. No significant differences in thrombin generation parameters were observed according to SIC status or SAC categories 24 h after ICU admission. ROTEM analyses demonstrated increased clot firmness together with elevated lysis indices and mildly prolonged EXTEM clotting times. Contact pathway-related markers, including factor XI, factor XII, and plasma kallikrein, were inversely associated with disease severity, while factor Xa activity decreased during the observation period.

**Conclusion:**

In this cohort of critically ill COVID-19 patients, thrombin generation parameters were inversely associated with disease severity, whereas no association was observed with SIC or SAC-defined coagulopathy. Viscoelastic testing demonstrated increased clot firmness and elevated lysis indices, while contact pathway-related markers decreased with increasing disease severity. These findings provide a descriptive characterization of coagulation abnormalities in critically ill COVID-19 patients and should be interpreted in the context of frequent ECMO support and systemic anticoagulation.

## Introduction

The coronavirus disease 2019 (COVID-19) pandemic has placed a significant burden on healthcare systems worldwide. Severe cases of COVID-19 leading to sepsis and acute respiratory distress syndrome (ARDS) often require the use of extracorporeal membrane oxygenation (ECMO) as a life-saving intervention. One factor associated with a poor prognosis was bleeding and thrombotic events [1, 2]. In conditions like sepsis, systemic immune activation - mediated by monocytes, endothelial cells, and neutrophils - can trigger widespread thrombosis and thromboembolism, eventually leading to consumptive coagulopathy and diffuse bleeding [3, 4]. In 2017, the international society on thrombosis and hemostasis introduced the sepsis-induced coagulopathy (SIC) score to facilitate the early bedside diagnosis of SIC, a distinct form of coagulopathy triggered by systemic inflammation [3]. The SIC score incorporates a simplified SOFA score together with platelet count and international normalized ratio [3]. Elevated SIC scores have been associated with increased morbidity and mortality in septic patients [4]. To classify the severity of coagulopathy into mild, moderate, and severe forms, the Sepsis-Associated Coagulopathy (SAC) score was developed [5].

Early in the pandemic, SARS-CoV-2 infection was found to cause marked coagulation activation due to endothelial injury and innate immune activation, a condition termed COVID-19-associated coagulopathy (CAC) [6]. Unlike traditional coagulopathies, CAC involves a complex interplay of hypercoagulability and inflammation that may progress to disseminated intravascular coagulation with diffuse bleeding, especially when aggravated by foreign surfaces and anticoagulation during ECMO therapy [7–9].

In critically ill COVID-19 patients, rotational thromboelastometry (ROTEM) provides insight into altered clot formation and reduced fibrinolytic activity, findings that have been associated with disease severity and adverse outcomes [10]. In addition, the thrombin generation assay (TGA) offers a dynamic and comprehensive assessment of coagulation, capturing both pro- and anticoagulant activity.

Recent studies investigating thrombin generation in COVID-19 have reported heterogeneous findings. While some studies described preserved or increased thrombin-generating capacity in patients with mild to moderate disease, others observed reduced endogenous thrombin potential and peak thrombin levels in critically ill patients, particularly in the context of systemic anticoagulation and organ failure [11–13]. These discrepancies may reflect differences in disease severity, anticoagulation strategies, timing of sampling, and methodological approaches [11–13]. Despite growing evidence, longitudinal data integrating thrombin generation assays, viscoelastic coagulation testing, and contact pathway-related markers in critically ill COVID-19 patients, particularly those requiring ECMO support, remain limited [14].

Therefore, the aim of this study was to provide a comprehensive description of coagulation abnormalities in critically ill patients with COVID-19 using thrombin generation assays, rotational thromboelastometry, and contact pathway-related markers, and to explore their association with disease severity over the first week of intensive care treatment.

## Materials and methods

### Study design and setting

This monocentric, prospective observational study was conducted between April 2020 and April 2021 in the intensive care unit (ICU) of the Department of Anesthesiology and Intensive Care Medicine at the University Hospital Essen, University of Duisburg-Essen, Germany. The study was conducted in accordance with the Declaration of Helsinki and was approved by the Ethics Committee of the Medical Faculty, University of Duisburg-Essen, Germany (Ethics Committee Number: 20-9242-BO, Approval Date: April 20, 2020). Additionally, the study was registered in the German Clinical Trials Register (Registration Number: DRKS-ID: DRKS00022841).

### Hospital settings

The University Hospital Essen is a tertiary care center. The ICU, operated by the Department of Anesthesiology and Intensive Care Medicine, is part of the West German Center for Infectious Diseases (WZI). Since the onset of the COVID-19 pandemic, the department has been treating critically ill SARS-CoV-2 patients as part of the University Hospital Essen’s medical care strategy. The clinic serves as a regional reference center for ARDS and specializes in ECMO therapy. A comprehensive description of our ARDS/ECMO unit and its structural features has already been presented by Herbstreit et al. [15].

### Patient recruitment

Patients were continuously enrolled in the study from April 2020 to April 2021. Inclusion criteria included a polymerase chain reaction (PCR)-confirmed SARS-CoV-2 infection, a SOFA score of more than 2, and age 18 years or older. Patients or their legal representatives provided written informed consent to participate in the study. For patients without legal representatives who were unable to provide consent, an independent medical reviewer approved study participation according to the guidelines of the Medical Faculty of the University of Duisburg-Essen. Upon recovery, these patients were retrospectively asked for their consent to participate in the study. Exclusion criteria included patient refusal to participate in the study, expected discharge from the ICU within 72 h of admission, pregnancy, a palliative care approach, and the patient’s imminent death.

### Patient treatment

All patients admitted to the ICU were treated according to a standardized treatment protocol [15]. We conducted global coagulation laboratory assessment, complemented by additional ROTEM analyses. A detailed description of patient care has already been presented by Herbstreit et al. [15]. The indication for vvECMO therapy followed the recommendations of the Extracorporeal Life Support Organization (ELSO) and was established in cases of failure of conservative therapy and/or cardiopulmonary deterioration [16]. Cannulation for vvECMO therapy was performed via bifemoral or femoro-jugular access.

The interdisciplinary approach to COVID-19 treatment was aligned with the most current therapeutic guidelines and scientific knowledge available between April 2020 and April 2021. In our study population, treatments included remdesivir, hydroxychloroquine, and convalescent plasma. All patients received systemic anticoagulation according to institutional ICU and vvECMO protocols using unfractionated heparin or argatroban. Anticoagulation was monitored using activated partial thromboplastin time (aPTT) with a target range of 40–50 s. Anti-Xa levels and detailed dosing data were not consistently available and were therefore not included in the analysis.For patients on vvECMO, platelet transfusions were standard practice when platelet counts fell below 50,000 per microliter. Additionally, acetylsalicylic acid (ASA) was routinely included as part of the general recommendations throughout the recruitment phase.

### Data acquisition

Demographic data and clinical parameters were collected as part of routine clinical practice. The Charlson Comorbidity Index (CCI) was used to categorize pre-existing conditions. Disease severity and organ dysfunction were assessed using the SOFA and SIC scores on days 1, 3, and 7 after ICU admission. The SOFA score was selected as a well-established measure of disease severity in critically ill ICU patients, including patients with viral sepsis. The modified SOFA score allowed us to calculate the SOFA even for patients whose neurological status was documented using the Richmond Agitation-Sedation Scale (RASS) instead of the Glasgow Coma Scale (GCS) [17]. For patients treated with vvECMO, we adjusted the SOFA score by adding an additional point. This modification increased the theoretical maximum SOFA score from 24 to 25 points. ARDS was defined according to the Berlin definition by the American-European Consensus Conference [18]. All patients were followed up according to MacIntyre’s recommendations for up to 28 days [19].

SIC score was defined according to Iba et al. [3] including the following three parameters: (1) platelet count, (2) international normalized ratio (INR), and (3) a truncated SOFA score comprising only respiratory, cardiovascular, hepatic, and renal subscores. Each parameter was scored from 0 to 2 points. A positive SIC score was defined by a total score ≥ 4 and a combined platelet (PSSC) and INR (ISSC) subscore ≥ 3 [3]. The SAC score categorizes coagulopathy into mild, moderate, or severe based on platelet count and international normalized ratio (INR). Specifically, mild SAC is defined as INR 1.2–1.4 and platelet count 100–150 × 10⁹/L; moderate SAC as INR 1.4–1.6 or platelet count 80–100 × 10⁹/L; and severe SAC as INR ≥ 1.6 or platelet count < 80 × 10⁹/L. This scoring system enables standardized assessment of coagulation abnormalities associated with sepsis [5].

### ROTEM analysis

To assess hemostatic function, rotational thromboelastometry (ROTEM^®^, Tem Innovations GmbH, Munich, Germany) was performed. Analyses were carried out in accordance with current manufacturer recommendations using citrated whole blood, processed within 30 min of collection. The standard assays EXTEM, INTEM, FIBTEM, and, if indicated, APTEM were employed to evaluate both the extrinsic and intrinsic coagulation pathways as well as fibrin polymerization and clot stability. All samples were analyzed using a ROTEM sigma^®^ device. Measured parameters included clotting time (CT), clot formation time (CFT), maximum clot firmness (MCF), and the lysis index at 30 min (LI30). Results were interpreted based on manufacturer-defined reference ranges.

### Thrombin generation assay

For thrombin generation analyses, blood samples were collected in sodium citrate tubes. Platelet-poor plasma (PPP) was prepared using a double-centrifugation protocol (2000 × g for 10 min followed by 4500 × g for 10 min), aliquoted, and stored at − 80 °C until analysis. Prior to testing, frozen plasma samples were thawed for 5 min at 37 °C.

Thrombin generation was assessed using the calibrated automated thrombogram (CAT) method. Briefly, PPP was incubated with trigger reagents containing tissue factor and phospholipids, and thrombin generation was initiated by addition of a fluorogenic substrate and calcium. Fluorescence was measured (excitation 390 nm, emission 460 nm) using an Infinite^®^ M1000 microplate reader (Tecan, Männedorf, Switzerland). Thrombin generation curves were analyzed using Thrombinoscope software (Thrombinoscope BV, Maastricht, The Netherlands). Lag time (LT), peak thrombin (PT), time-to-peak (TTP), and endogenous thrombin potential (ETP) were recorded.

### Statistical analyses

All collected data were systematically recorded in a digital database using Microsoft Access (Microsoft Corporation, Redmond, WA, USA). Subsequent statistical analyses were performed with GraphPad Prism version 9.0 for Mac (GraphPad Software, La Jolla, CA, USA; accessed on July 18, 2022, via http://www.graphpad.com).

Given the exploratory nature of the study and the limited sample size, analyses were primarily descriptive and exploratory. Repeated longitudinal measurements were assessed using exploratory correlation analyses and descriptive longitudinal comparisons rather than formal longitudinal mixed-effects models. No formal correction for multiple testing was applied. Secondary and subgroup analyses should therefore be considered exploratory.

Descriptive statistics are expressed as medians and interquartile ranges (IQR). Normality was assessed using the D’Agostino–Pearson test. Comparisons between two independent groups were performed using the Mann–Whitney U test. Associations between variables were evaluated using Spearman’s rank correlation coefficient. Statistical significance was defined as a two-sided p-value < 0.05.

Exploratory correlation analyses were performed to assess the relationship between endogenous thrombin potential (ETP) and other coagulation-related parameters, including ROTEM-derived variables, factor XIIa, factor Xa activity, fibrinogen, aPTT, and INR.

## Results

### Characteristics of critically ill COVID-19 patients

A total of 48 patients were included in the study. The median age of the cohort was 59 years (interquartile range (IQR): 51–65) and 34 (71%) patients were male. The clinical characteristics revealed a median CCI of 2 (IQR: 2–3), and a median body mass index (BMI) of 31 kg/m² (IQR: 28–37). On admission to the ICU 6 patients (13%) were spontaneously breathing with oxygen supply, and 42 patients (87%) were ventilated (5 patients with non-invasive ventilation and 37 patients with invasive ventilation). Additionally, 38 patients (79%) required vvECMO during their ICU stay. 16 patients (33%) survived at 28 days. The median SOFA score remained stable around 13, while the number of patients with a positive SIC score was consistently 12.5% across all days. Table 1 summarizes the demographic, clinical, and outcome parameters. Descriptive data on thromboembolic and bleeding events are provided in the supplementary material (Supplementary Table S1).

**Table 1:** Clinical data, outcome and disease severity of critically ill COVID-19 patients (*n* = 48); Values are expressed as # median (interquartile range). The number in brackets [ ] shows the number of patients with missing data. CCI— Charlson Comorbidity Index; BMI—Body mass index; vvECMO—venovenous extracorporeal membrane oxygenation; SOFA—Sequential Organ Failure Assessment Score; SIC— Sepsis-Induced Coagulopathy Score; SAC— Sepsis associated Coagulopathie Score

Characteristics of critically ill patients with COVID-19 (*n* = 48)			
*clinical data*			
age [in years] #			59 (51–65)
male			34 (71%)
CCI #			2 (2–3)
BMI [in kg/m²] #			31 (28–37)
at ICU admission			
spontaneous breathing, oxygen dependent			6 (13%)
ventilated			42 (87%)
non-invasive			5 (10%)
invasive			37 (77%)
vvECMO during ICU stay			38 (79%)
initiation at referring ICU			34 (71%)
initiation after ICU admission			4 (8%)
*outcome*			
28-day survivors			16 (33%)
*disease severity*			
	*day 1*	*day 3*	*day 7*
SOFA Score #	13 (10–15) [2]	13 (10–15) [3]	13 (11–16) [7]
SIC Score positive	6 (12.5%)	6 (13.6%)	6 (13.6%)
SAC Score			
mild	0	2 (4.2%)	0
moderate	6 (12.5%)	7 (14.6%)	8 (16.7%)
severe	4 (8.3%)	8 (16.7%)	12 (25.0%)

### Thrombin generation is associated with disease severity in COVID-19 patients, but not with the severity of coagulopathy

**Fig. 1:**
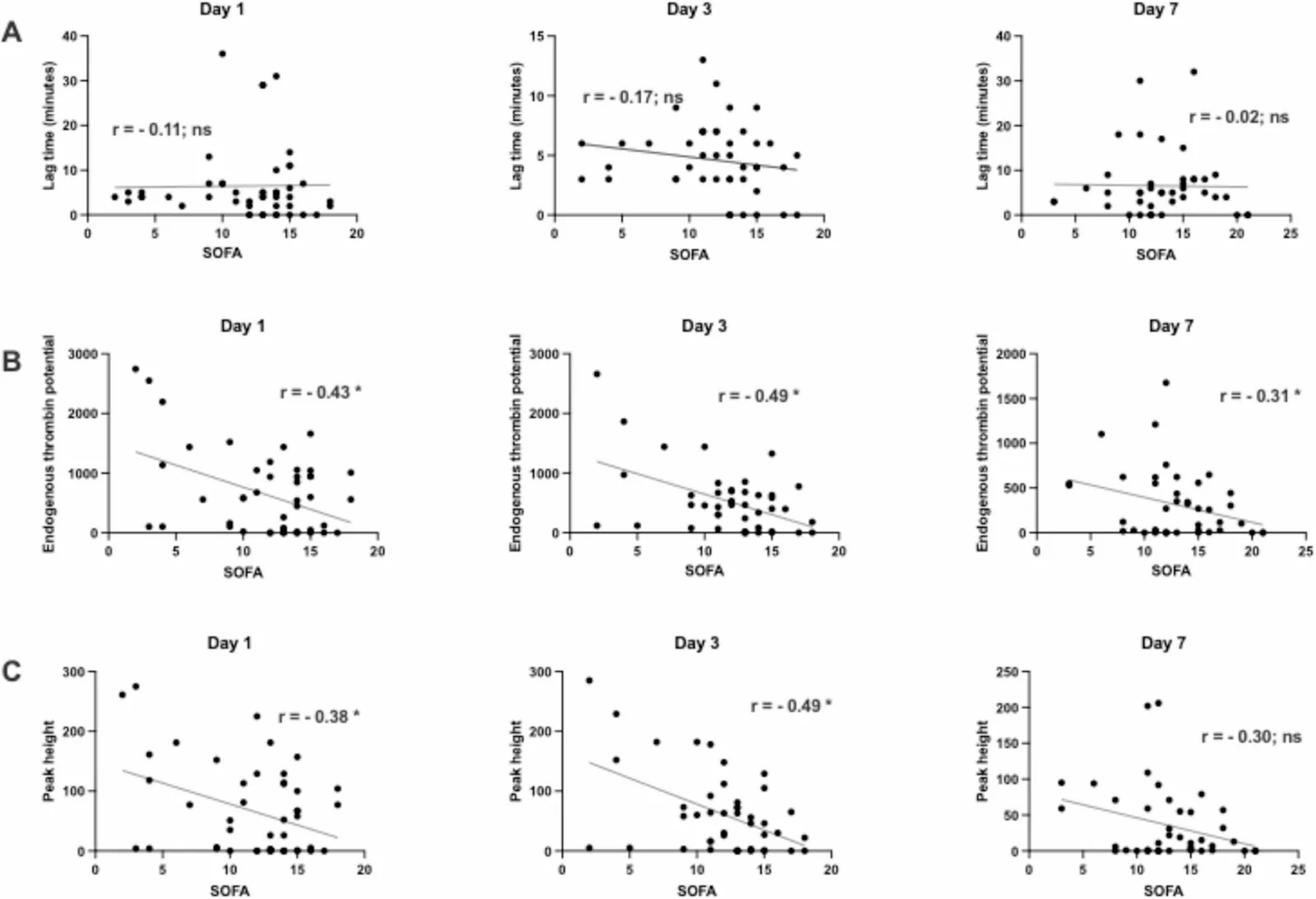
Relationship between thrombin generation assay (TGA) parameters and disease severity, assessed by the Sequential Organ Failure Assessment (SOFA) score. Panel **A** shows lag time (LT), panel **B** endogenous thrombin potential (ETP), and panel **C** peak thrombin measured on days 1 (*n* = 46), 3 (*n* = 45), and 7 (*n* = 41) after ICU admission. Correlations were assessed using Spearman’s rank correlation coefficient. Significant inverse correlations between ETP and SOFA score were observed on days 1, 3, and 7. ns, not significant

To address the primary objective of this study, we evaluated the relationship between TGA on day 1, 3, and 7 after admission to ICU and disease severity using the SOFA score (Fig. 1). Lag time (LT) did not correlate with SOFA scores at any time point (day 1: *r* = − 0.11; day 3: *r* = − 0.17; day 7: *r* = − 0.02). In contrast, endogenous thrombin potential (ETP) showed a consistent inverse correlation with SOFA scores across all days (day 1: *r* = − 0.43, 95% CI: -0.63 to -0.17, *p* = 0.0021; day 3: *r* = − 0.49, 95% CI: -0.68 to -0.22, *p* = 0.0169; day 7: *r* = − 0.31, 95% CI: -0.56 to -0.01, *p* = 0.0468). Peak thrombin was also inversely correlated with SOFA scores on day 1 (*r* = − 0.38; 95% CI: -0.59 to -0.10, *p* = 0.0081) and day 3 (*r* = − 0.49; 95% CI: -0.68 to -0.22, *p* = 0.0007), whereas no significant correlation was observed on day 7 (*r* = − 0.30).

Next, we examined the relationship between coagulopathy severity, as assessed by the SIC and SAC scores. No significant differences in LT, ETP, or peak thrombin were observed between SIC-positive and SIC-negative patients or across SAC severity categories 24 h after ICU admission (Table S2).

### Temporal evolution of endogenous thrombin potential in patients with severe COVID-19

**Fig. 2:**
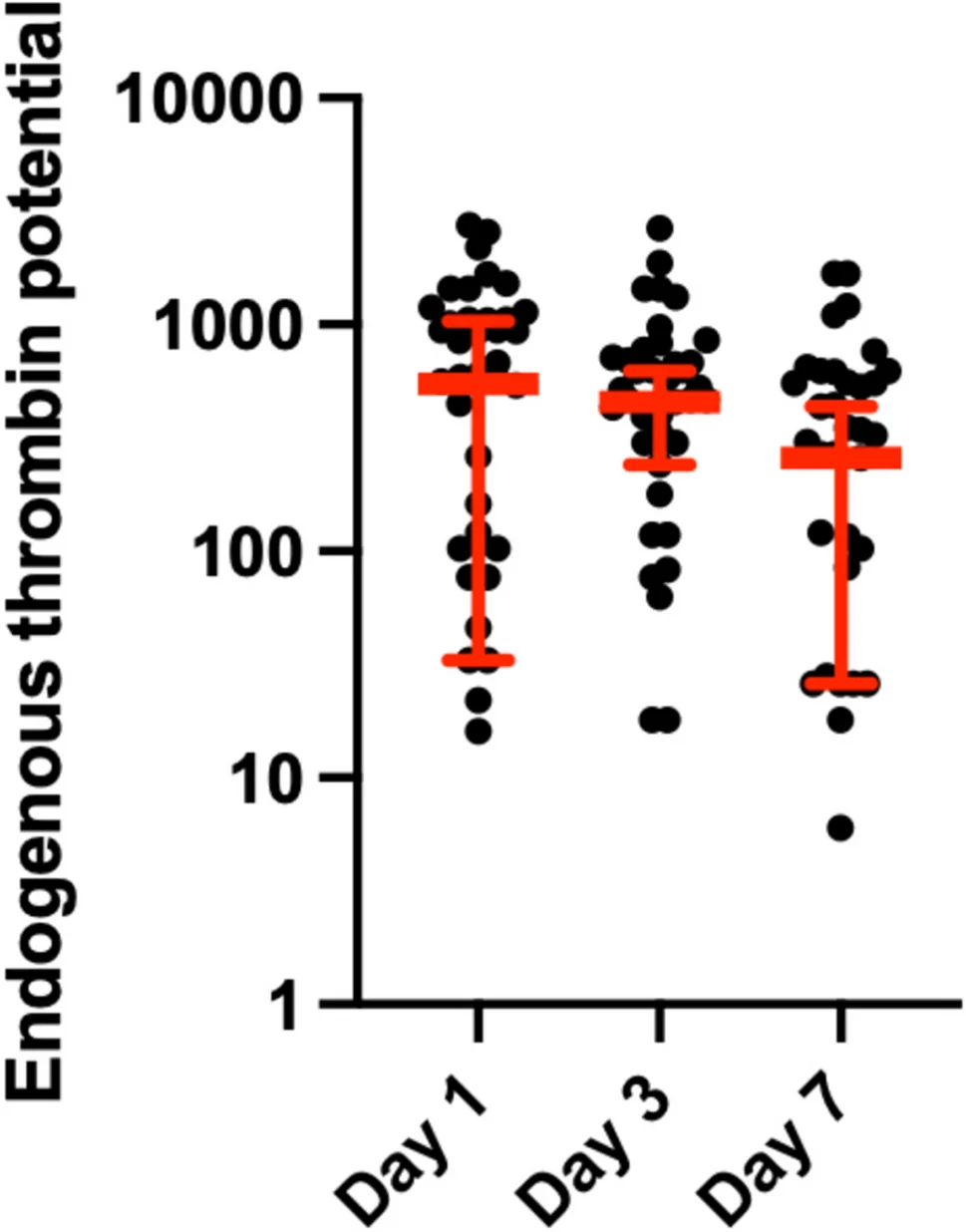
Endogenous thrombin potential (ETP) was measured on days 1 (*n* = 48), 3 (*n* = 45), and 7 (*n* = 43) in patients with severe COVID-19. Individual data points are shown together with median and interquartile range (IQR). Median ETP values were 549.5 (33.0–1035.0), 456.0 (80.0–686.5), and 258.0 (6.0–551.0) on days 1, 3, and 7, respectively

**Fig. 3:**
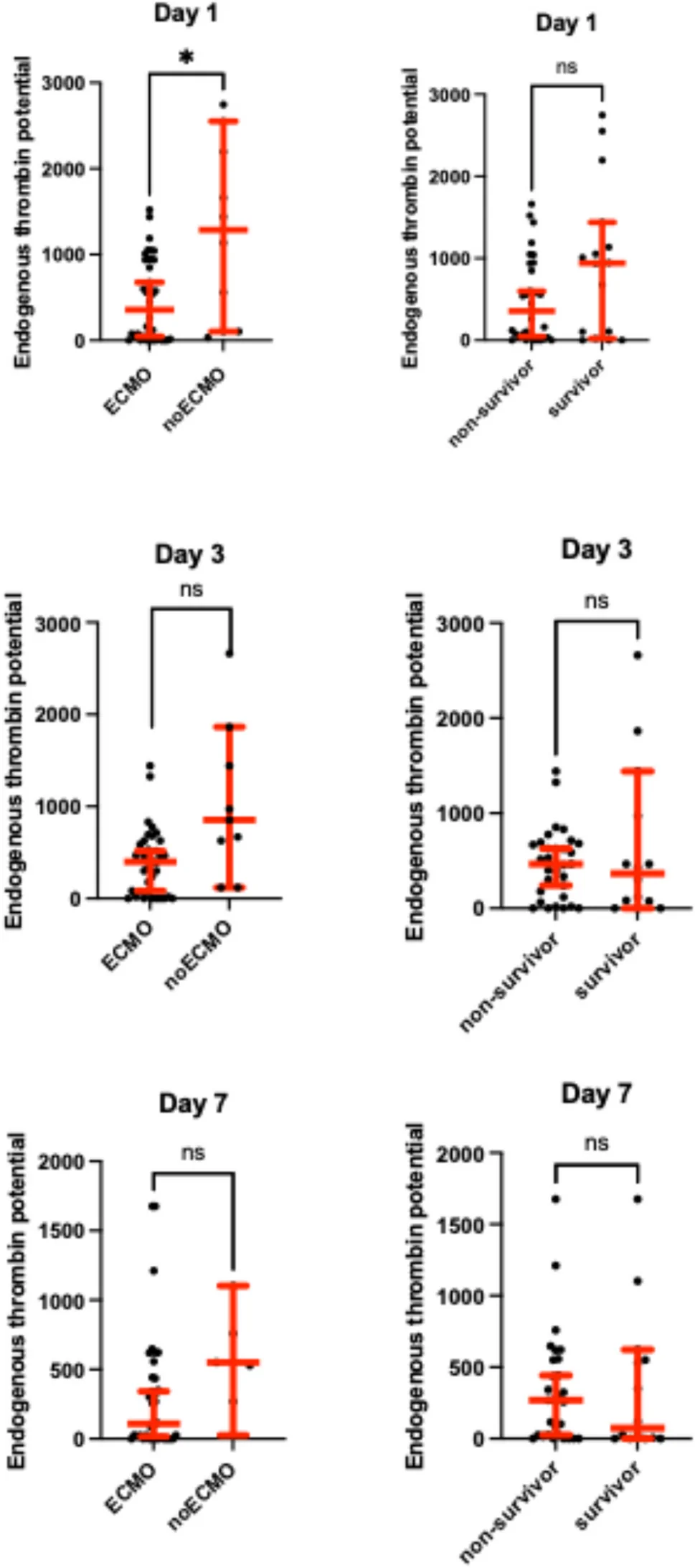
Endogenous thrombin potential (ETP) measured on days 1, 3, and 7 in patients with severe COVID-19. Left panels show comparisons between patients receiving extracorporeal membrane oxygenation (ECMO) support and patients without ECMO support. Right panels show comparisons between survivors and non-survivors. Individual data points are displayed together with median and interquartile range. A statistically significant difference between ECMO and non-ECMO patients was observed on day 1 (*p* = 0.0125), whereas no significant differences were detected on days 3 or 7. No significant differences in ETP were observed between survivors and non-survivors at any assessed time point. ns, not significant

To provide a descriptive overview of ETP dynamics during the observation period, serial ETP measurements obtained on days 1, 3, and 7 were analyzed. In the overall cohort, median ETP values were 549.5 on day 1 (*n* = 48), 456.0 on day 3 (*n* = 45), and 258.0 on day 7 (*n* = 43). No statistically significant differences in ETP were observed between survivors and non-survivors at any assessed time point. In exploratory analyses stratified by ECMO status, ETP values were lower in patients receiving ECMO support than in patients without ECMO support on day 1 (median 355.5 vs. 1287; *p* = 0.0125), whereas no significant differences were observed on days 3 and 7. The corresponding distributions of ETP values over time and according to survival and ECMO status are shown in Figs. 2 and 3.

To assess the relationship between TGA and other coagulation-related measures 24 h after ICU admission, Spearman correlation analyses were performed between ETP and ROTEM parameters, coagulation activation markers, and routine coagulation tests (Table S3). ETP was not significantly associated with INTEM or EXTEM CT or MCF, with correlation coefficients ranging from − 0.076 to 0.230 (all *p* > 0.13). Likewise, no significant correlation was observed between ETP and factor XIIa levels (ρ = 0.034, *p* = 0.822), fibrinogen concentration (ρ = −0.020, *p* = 0.877), or INR (ρ = 0.071, *p* = 0.607). In contrast, ETP showed a moderate positive correlation with factor Xa activity (ρ = 0.634, 95% CI 0.417–0.783; *p* < 0.0001) and a moderate inverse correlation with aPTT (ρ = −0.407, 95% CI − 0.607 to − 0.103; *p* = 0.007).

### ROTEM demonstrates increased clot firmness and reduced fibrinolytic activity in critically ill COVID-19 patients

**Fig. 4:**
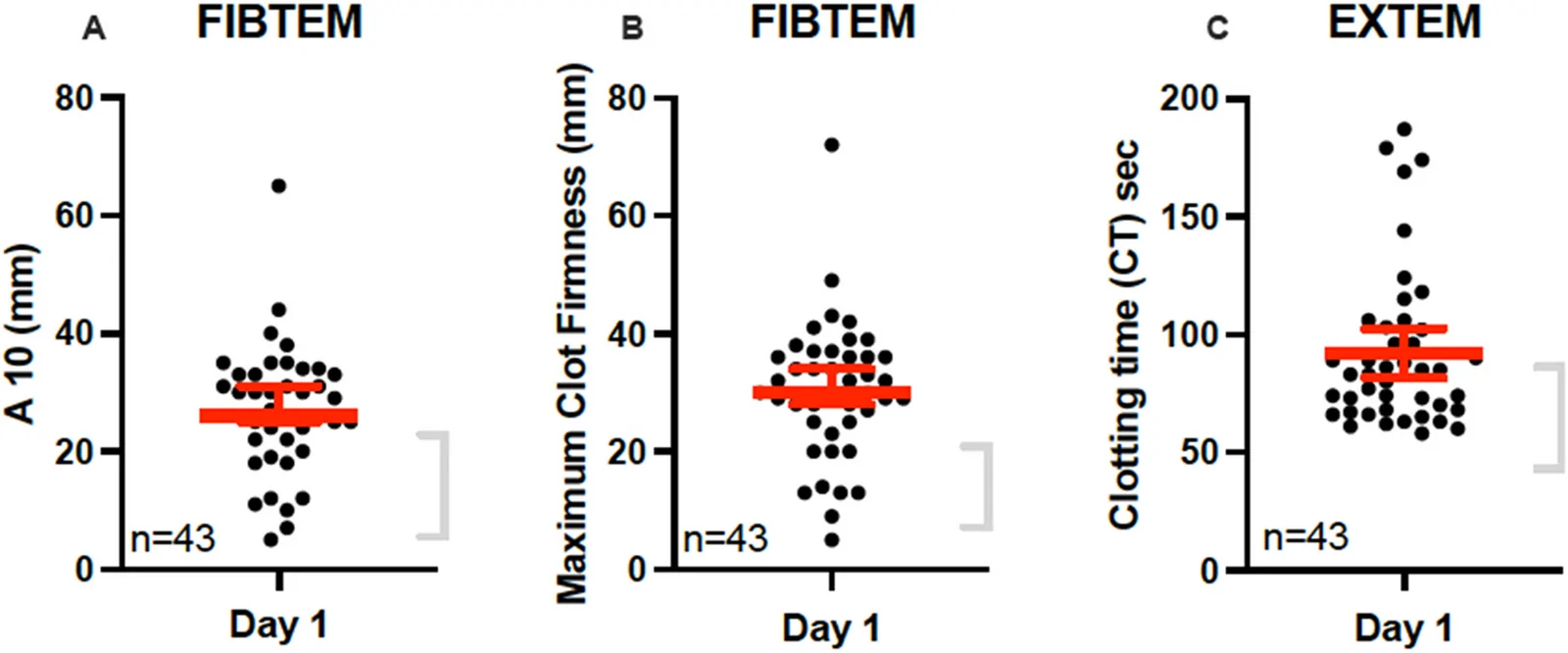
ROTEM parameters on day 1 (*n* = 43). Figures **A**–**C** display median values with interquartile ranges (IQR); reference ranges are shown in gray. FIBTEM demonstrated increased clot strength, reflected by elevated amplitude at 10 min (A10 [mm]: 26 [20–33] vs. reference range 7–23) and increased maximum clot firmness (MCF [mm]: 30 [25–36] vs. 9–25) (**A**, **B**), consistent with increased fibrinogen-dependent clot strength. Clot stability was increased, indicating reduced fibrinolysis, as shown by the lysis index at 30 min in EXTEM (LI30 [%]: 100 [100–100]). Coagulation initiation was not accelerated, with only mildly prolonged clotting time in EXTEM (CT [s]: 83 [68–103] vs. 38–79) (**C**). A10, amplitude at 10 min; MCF, maximum clot firmness; LI30, lysis index at 30 min; CT, clotting time

The results of 43 rotational thromboelastometry (ROTEM) analyses performed on day 1 demonstrated a heterogeneous viscoelastic coagulation profile in patients with COVID-19 (Fig. 4). Increased clot firmness was observed in the FIBTEM assay, reflected by elevated amplitude at 10 min (A10: median 26 mm, IQR 22–33 mm; reference range: 7–23 mm) and increased maximum clot firmness (MCF: median 30 mm, IQR 25–36 mm; reference range: 9–25 mm). These findings are consistent with increased fibrinogen-dependent clot strength rather than direct evidence of global hypercoagulability. In addition, clot stability was increased, reflected by a lysis index at 30 min (LI30) of 100% (IQR 100–100%) in all patients, suggesting reduced fibrinolytic activity. Clotting time in the EXTEM assay was mildly prolonged (CT: median 83 s, IQR 68–103 s; reference range: 38–79 s), indicating delayed coagulation initiation despite increased clot firmness. No significant changes in ROTEM parameters were observed over the course of treatment. A systematic presentation of the ROTEM results and the laboratory coagulation parameters from days 1, 3, and 7 are presented in Table S4 in the appendix.

### Association of coagulation factors XI, XII and plasma kallikrein with disease severity and mortality in critically Ill COVID-19 patients

**Fig. 5:**
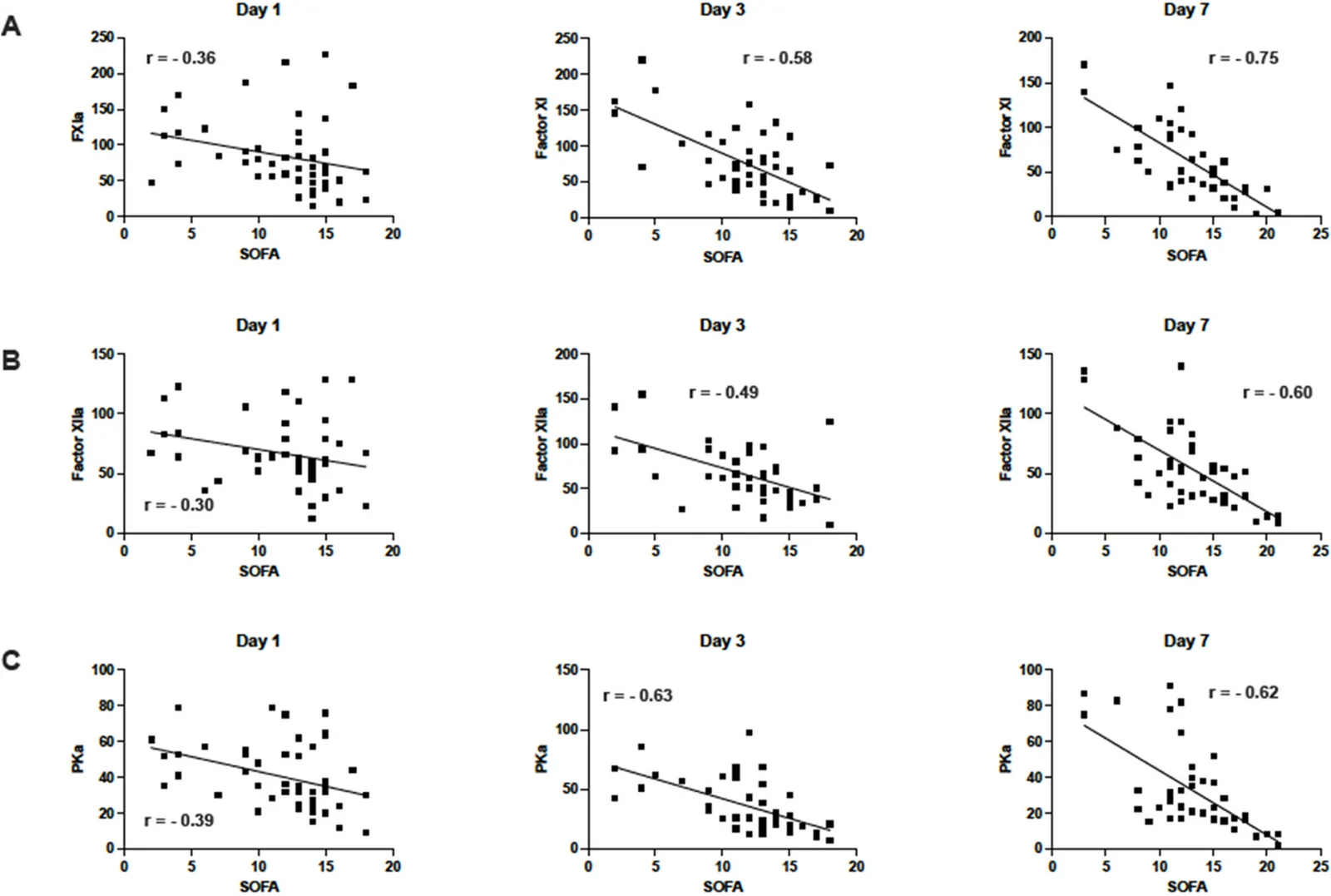
Association of factor XI (**A**), factor XII (**B**), and plasma kallikrein (**C**) with disease severity in COVID-19 patients, assessed by the Sequential Organ Failure Assessment (SOFA) score. Measurements were performed on days 1 (*n* = 46), 3 (*n* = 45), and 7 (*n* = 41) after ICU admission. Significant inverse correlations between all three contact pathway-related markers and SOFA score were observed across the study period, with the strongest associations on day 7 (factor XI: *r* = − 0.75, 95% CI − 0.86 to − 0.57; factor XII: *r* = − 0.60, 95% CI − 0.77 to − 0.35; plasma kallikrein: *r* = − 0.62, 95% CI − 0.78 to − 0.38; all *p* = 0.0001). Correlations were assessed using Spearman’s rank correlation coefficient

With increasing disease severity, levels of factor XI, factor XII, and plasma kallikrein decreased and were consistently associated with SOFA scores on days 1, 3, and 7 after ICU admission. The strongest correlations for all three factors were observed on day 7 (factor XI *r*= -0.75, 95% CI: -0.86 to -0.57, *p* = 0.0001 ; factor XII *r*= -0.60, 95% CI: -0.77 to -0.35, *p* = 0.0001; plasma kallikrein *r*= -0.62, 95% CI: -0.78 to -0.38, *p* = 0.0001; Fig. 5). Exploratory analyses revealed no significant differences in factor XI, factor XII, or plasma kallikrein levels between patients with and without ECMO support at any assessed time point.

**Fig. 6:**
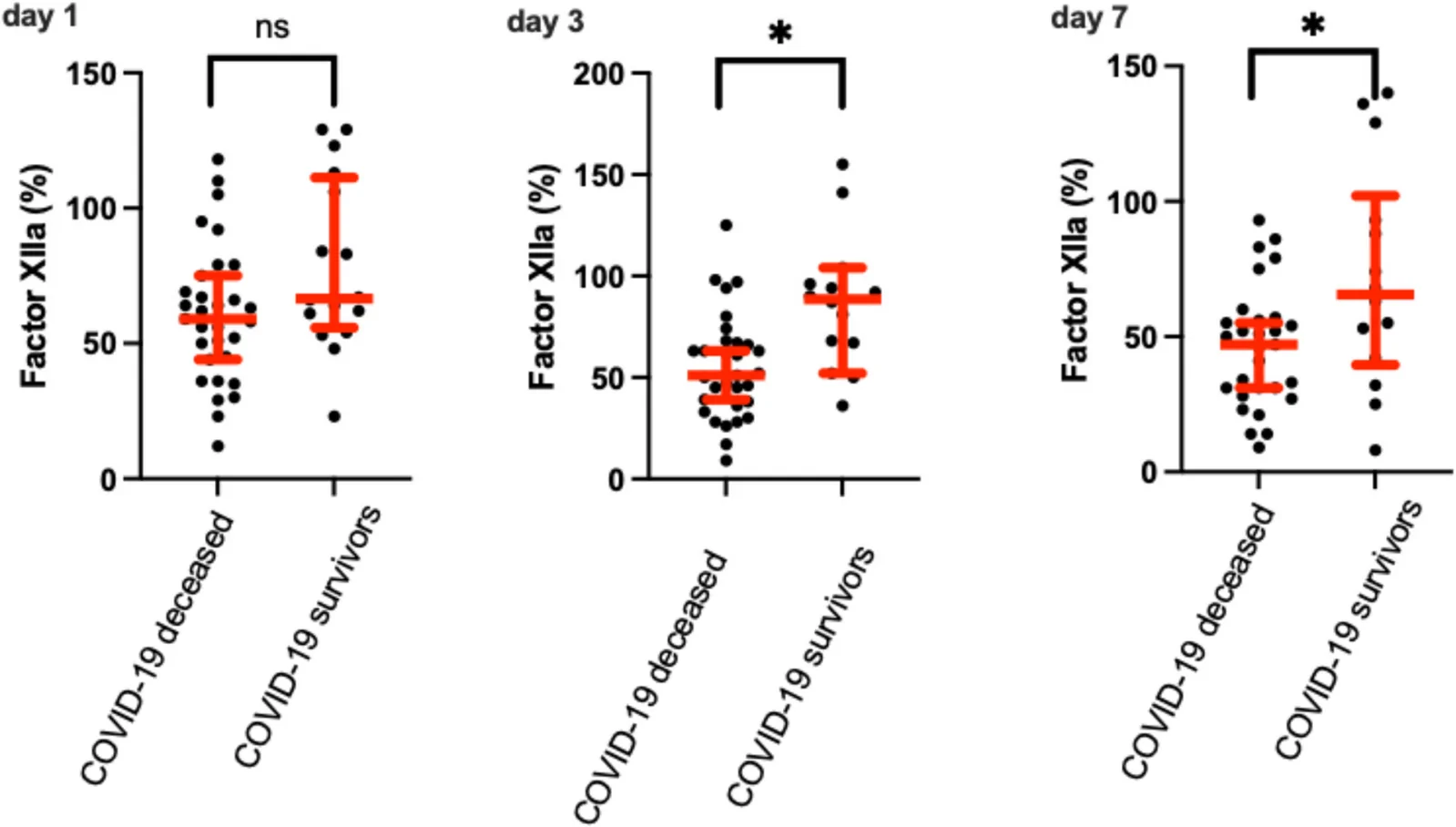
Factor XIIa (%) levels were significantly lower in critically ill COVID-19 patients who died within 28 days compared with survivors on day 3 (51.0 (*n* = 31) vs. 88.5 (*n* = 14); *p* = 0.0016) and on day 7 (47.0 (*n* = 29) vs. 65.5 (*n* = 14); *p* = 0.038) after ICU admission. No significant difference was observed on day 1. ns, not significant

Moreover, factor XIIa was significantly lower on day 3 and 7 after admission on ICU in patients who died within 28 days compared to survivors (on day 3: 51.0 (*n* = 31) vs. 88.5 (*n* = 14); *p* = 0.0016 and on day 7: 47.0 (*n* = 29) vs. 65.5 (*n* = 14); *p* = 0.038; Fig. 6).

### Factor Xa decreases in critically ill patients with COVID-19 within the first 7 days after ICU admission

**Fig. 7:**
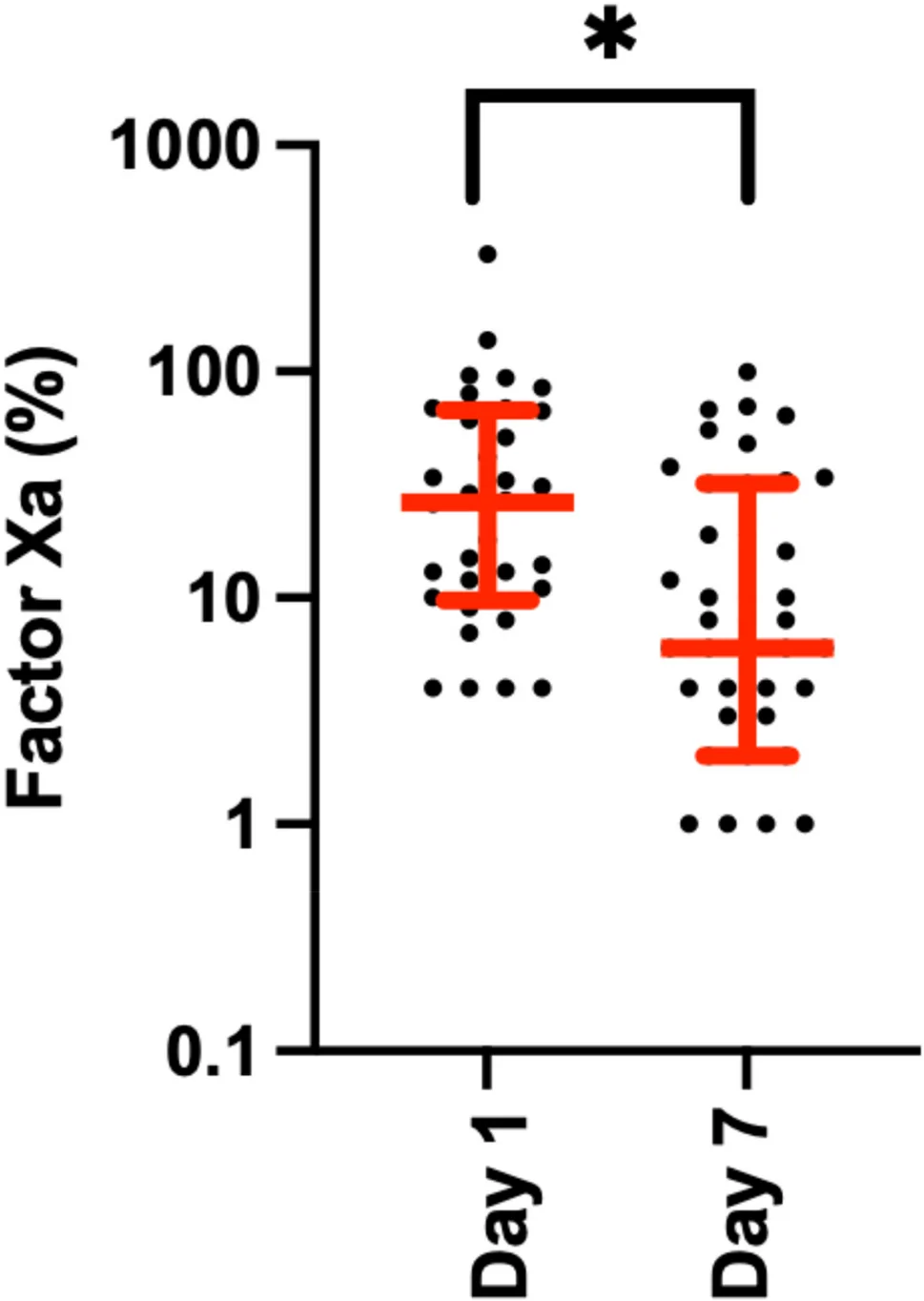
Factor Xa activity (%) in critically ill COVID-19 patients on days 1 and 7 after ICU admission. Scatterplots display median factor Xa activity levels on day 1 (26.50%; *n* = 34) and day 7 (6.00%; *n* = 43). *p = 0.0009

Factor Xa decreased significantly over the course of the ICU stay from admission to day 7 (day 1: 26.5%, *n* = 34 vs. day 7: 6.0%, *n* = 43; *p* = 0.0009; Fig. 7). However, this finding may also be influenced by systemic anticoagulation. In contrast, no relevant changes in factor II levels were observed during the first 7 days after ICU admission.

## Discussion

In this prospective single-center observational study, we investigated the association between thrombin generation assay (TGA) parameters and disease severity in critically ill COVID-19 patients. We observed an inverse relationship between TGA parameters and SOFA scores, reflected by both ETP and PT, which may be consistent with lower thrombin-generating capacity in patients with more severe organ dysfunction. The observed coagulation profile may be influenced by the combined effects of COVID-19-associated coagulopathy, extracorporeal support, critical illness, and systemic anticoagulation. Rather than identifying a distinct isolated mechanism, our study provides a longitudinal descriptive assessment of thrombin generation, viscoelastic coagulation parameters, and contact pathway-associated markers in critically ill COVID-19 patients.

The relationship between TGA and disease severity in COVID-19 remains incompletely understood, with previous studies reporting heterogeneous results. Our findings are in line with previous reports. For example, Campello et al. reported significantly reduced ETP levels in ICU patients with severe COVID-19 compared with patients with milder disease and healthy controls [20]. In contrast, other studies have found no clear association between TGA parameters and clinical outcomes. For example, Cohen et al. reported no significant differences in TGA between survivors and non-survivors in a cohort of hospitalized COVID-19 patients [21]. Similarly, the study by de la Morena-Barrio et al. demonstrated that individual TGA parameters were not independently associated with mortality, although the D-dimer/ETP ratio showed prognostic relevance [22]. Our findings partly support these observations, suggesting that while thrombin generation may reflect disease severity, it may not reliably distinguish between different degrees of coagulopathy or predict clinical outcomes when used in isolation. Interestingly, TGA did not differ between SIC-positive and SIC-negative patients or across SAC severity categories. This observation may reflect the distinct characteristics of CAC, which differs in several aspects from classical SIC. Previous studies have suggested that CAC represents a unique form of immunothrombosis characterized by hypercoagulability, endothelial activation, and dysregulated inflammatory responses rather than the consumptive coagulopathy typically observed in disseminated intravascular coagulation (DIC) [23, 24]. In addition, the applicability and interpretation of SIC/SAC scoring systems in critically ill COVID-19 patients may be further limited by systemic anticoagulation and frequent vvECMO support.

The viscoelastic profile observed in our cohort suggests a heterogeneous coagulation phenotype rather than a uniformly hypercoagulable state. Increased clot firmness may be related to elevated fibrinogen concentrations during the acute phase and was accompanied by elevated EXTEM lysis indices, a pattern that may be compatible with reduced fibrinolytic activity as previously reported in critically ill patients with COVID-19 [25].

Previous ROTEM studies in COVID-19 have characterized hypercoagulability using broader viscoelastic profiles rather than isolated FIBTEM-derived parameters. For example, Calvet et al. defined hypercoagulability using a composite ROTEM-based approach that incorporated EXTEM clot formation time, clot firmness, clot amplitude, fibrinolysis parameters, and clot elasticity indices [26]. Similarly, Loutsidi et al. assessed hypercoagulable ROTEM profiles using multiple viscoelastic parameters and demonstrated associations with persistent pulmonary dysfunction and post-COVID sequelae [27]. Furthermore, van de Berg et al. demonstrated that fibrinogen concentrations and anticoagulation substantially influence ROTEM-derived clot firmness and thrombin generation, highlighting the importance of interpreting viscoelastic findings within the broader context of global coagulation assays [12]. In this context, our findings are most consistent with increased fibrinogen-dependent clot strength and reduced fibrinolytic activity. However, the concomitant presence of mildly prolonged EXTEM clotting times and reduced thrombin generation parameters does not support a uniform hypercoagulable phenotype. Instead, these findings suggest a heterogeneous coagulation profile that may be influenced by critical illness, systemic anticoagulation, and the high prevalence of ECMO support in our cohort.

In contrast, prolonged EXTEM clotting time and reduced thrombin generation were also observed despite increased clot firmness. As ROTEM-derived parameters and thrombin generation assays assess different aspects of haemostasis, these findings should not necessarily be interpreted as contradictory but may provide complementary information on the coagulation profile of critically ill patients with COVID-19.

Fibrinolytic capacity was assessed using conventional EXTEM lysis indices (LI30 and LI60) only. However, these parameters have limited sensitivity for the detection of hypofibrinolysis. More sensitive approaches, including tPA-augmented viscoelastic testing, may allow improved characterization of fibrinolytic phenotypes and should be considered in future studies [28]. FIBTEM-derived clot firmness primarily reflects fibrinogen-dependent clot properties and should not be interpreted as a direct measure of global coagulation activation or hypercoagulability.

ROTEM-derived parameters and TGA reflect different dimensions of hemostasis. While ROTEM primarily assesses viscoelastic clot formation and clot firmness in whole blood, TGA evaluates plasma thrombin-generating capacity under ex vivo conditions. Accordingly, the observed combination of increased clot firmness and reduced ETP should not be interpreted as contradictory findings but rather as reflecting different aspects of coagulation that may additionally be influenced by anticoagulation and ECMO support. Exploratory correlation analyses of coagulation parameters obtained 24 h after ICU admission demonstrated no significant associations between ETP and ROTEM-derived parameters, factor XIIa, fibrinogen, or INR, whereas significant correlations were observed with factor Xa activity and aPTT. These findings provide additional context for the interpretation of thrombin generation in critically ill patients with COVID-19. Taken together, these findings suggest that increasing disease severity was associated with a progressive reduction in measurable thrombin-generating capacity and alterations in coagulation-related biomarkers, despite preserved or increased clot firmness in viscoelastic testing. This pattern highlights the complex and multifaceted nature of COVID-19-associated coagulopathy in critically ill patients and underscores that different coagulation assays may reflect distinct aspects of haemostatic dysfunction.

An important observation of our study is the association between disease severity and components of the contact activation pathway, including factor XI, factor XII, and plasma kallikrein. Levels of these factors were inversely correlated with SOFA scores, which may reflect alterations in contact pathway-related markers in patients with more severe disease. These findings are consistent with previous studies highlighting the role of the kallikrein–kinin system in COVID-19–associated thromboinflammation. Lipcsey et al. demonstrated that activation of the kallikrein–kinin system was linked to increased mortality and worse clinical outcomes in critically ill COVID-19 patients [29]. Furthermore, reduced factor XII activity has been described in patients receiving ECMO therapy, which may influence coagulation dynamics in critically ill patients [30]. In addition, factor XII activation has been associated with neutrophil extracellular traps (NETs) in COVID-19 lung tissue, further supporting the involvement of contact system activation in the pathophysiology of COVID-19-associated coagulopathy [31]. Our results are in line with previous observations and are compatible with alterations in contact pathway-related markers. The observed decrease in factor XI, factor XII, and plasma kallikrein levels with increasing disease severity may be compatible with alterations in contact pathway-related markers in patients with more severe disease. However, given the high proportion of patients receiving ECMO support and systemic anticoagulation, the underlying mechanisms cannot be determined from the present observational data.

We also observed a significant decline in factor Xa levels during the first week of ICU treatment. Although longitudinal data on individual coagulation factor dynamics in critically ill patients remain limited, several reports have described alterations in factor Xa activity in patients with severe COVID-19 [32]. Declining factor Xa activity observed in this cohort may at least partly reflect the effects of systemic anticoagulation rather than disease-specific biological alterations. Notably, longitudinal data on individual coagulation factors in non-COVID critically ill patients are scarce [33]. Thus, the observed decline in factor Xa may represent a phenomenon that is particularly pronounced or more readily detectable in severe COVID-19, although this remains speculative.

Several important limitations need to be considered when interpreting our findings. First, substantial confounding is introduced by anticoagulation and extracorporeal support. All patients received therapeutic anticoagulation, and the majority required vvECMO, both of which are known to significantly affect coagulation parameters. Heparin directly inhibits factor Xa and influences thrombin generation assays, while ECMO circuits can activate the contact pathway and lead to consumption of factor XII and related components. Consequently, several of our observations—particularly the decline in factor Xa activity and alterations in thrombin generation—may at least partly reflect treatment-associated effects rather than disease-specific alterations. Given the observational study design, the individual contributions of COVID-19-associated coagulopathy, anticoagulation, and extracorporeal support cannot be fully disentangled. In addition, the lack of standardized timing of blood sampling in relation to anticoagulant administration and the absence of anti-Xa measurements further limit interpretability. Second, the heterogeneity of disease stage at study inclusion represents a key limitation. Many patients were transferred from external hospitals at advanced stages of critical illness, likely contributing to the high severity of disease observed in this cohort. As a result, the time point defined as “day 1” does not reflect a uniform onset of critical illness but rather a variable and often advanced disease stage. This limits the interpretation of temporal changes in coagulation parameters, which may reflect differences in disease stage rather than true longitudinal dynamics. Some laboratory parameters, including D-dimer measurements, were not available for all time points and analyses were therefore based on available data. Furthermore, analyses stratified by respiratory support modalities and compliance-based ARDS phenotypes were limited by the small subgroup sizes. Given the exploratory study design, relatively small sample size, repeated measurements, and multiple statistical comparisons, findings should be interpreted cautiously and considered hypothesis-generating. Furthermore, the study cohort represents a highly selected population of critically ill COVID-19 patients treated at a tertiary referral center, with frequent vvECMO support and advanced disease severity. Consequently, the generalizability of these findings to broader ICU populations, later stages of the pandemic, or other viral diseases may be limited.

Despite these largely confirmatory findings, our study provides an integrative perspective by combining longitudinal assessment of TGA, rotational thromboelastometry analysis, and contact pathway factors within a single cohort of critically ill patients. This approach allows for a more comprehensive characterization of coagulation dynamics in advanced disease stages, particularly in a population with a high prevalence of ECMO support. To our knowledge, few studies have simultaneously evaluated these complementary aspects of coagulation in such a severely ill and treatment-intensive cohort. In this context, our findings highlight the complexity of interpreting coagulation parameters under conditions of substantial therapeutic intervention and underscore the importance of considering treatment-related confounders when assessing COVID-19–associated coagulopathy. The findings of this study primarily reflect coagulation profiles in a highly selected cohort of critically ill COVID-19 patients receiving systemic anticoagulation and frequent vvECMO support and may therefore not be generalizable to broader ICU populations or later stages of the pandemic.

Thus, the primary contribution of our study lies not in the identification of novel individual biomarkers, but in the integrated and longitudinal evaluation of established coagulation parameters in a highly selected population of critically ill patients.

## Conclusion

In this prospective observational cohort of critically ill COVID-19 patients, thrombin generation parameters were inversely associated with disease severity during the first week after ICU admission. In addition, viscoelastic testing demonstrated increased clot firmness and elevated lysis indices, while contact pathway-related markers showed inverse associations with disease severity. Together, these findings provide a descriptive overview of coagulation abnormalities in a severely ill COVID-19 population. Given the high prevalence of ECMO support and systemic anticoagulation, the observed associations should be interpreted cautiously and do not permit conclusions regarding underlying mechanisms or causality.

## Data Availability

The datasets used and/or analysed during the current study are available from the corresponding author on reasonable request.
