# Correction: Thrombin generation, viscoelastic coagulation profiles, and disease severity in critically Ill COVID-19 patients: a prospective observational study

**DOI:** 10.1186/s12871-026-04297-7

**Published:** 2026-10-10

**Authors:** Florian Espeter, Lena Garczarek, David Künne, Henning Kuhlmann, Stefan Heitmeier, Thomas Schmoch, Thorsten Brenner, Karsten Schmidt

**Affiliations:** 1 Department of Anesthesiology and Intensive Care Medicine, University Hospital Essen, University Duisburg-Essen, 45147 Essen, Germany; 2 Bayer AG, Research and Development Pharmaceuticals, 42117 Wuppertal, Germany; 3 Departement of Anesthesiology and Intensive Care Medicine, Hôpitaux Robert Schuman- Hôpital Kirchberg, 9, Rue Edward Steichen, Luxembourg City, 2540 Luxembourg

**Correction: BMC Anesthesiol. 2026;26:485**

https://doi.org/10.1186/s12871-026-04166-3.

Following publication of the original article [1], the author identified an error in the name of the device used in the article. ROTEM delta should be ROTEM sigma.

This correction does not affect the data, analyses, results and conclusions of the article.

The original article has been updated.
